# Scientific integrity in research methods

**DOI:** 10.3389/fpsyg.2015.01562

**Published:** 2015-11-03

**Authors:** Jordan R. Schoenherr

**Affiliations:** Department of Psychology, Carleton UniversityOttawa, ON, Canada

**Keywords:** scientific integrity, ethics, research methods, implicit curriculum, research misconduct

## Introduction

Recent cases of research misconduct have prompted psychologists to suggest that there is too much vulnerability in the research process (e.g., Simmons et al., 2011; Pashler and Harris, 2012). Regardless of whether this is the case, ensuring the integrity of a discipline requires a clear understanding of what conventions and norms define the research process. In what follows, I consider the research integrity curriculum of North American psychology. In particular, I will claim that a major impediment to ensuring responsible research practices is an underspecified and understudied curriculum.

## Defining curriculum

A general distinction made in the education literature is that between the explicit curriculum and implicit curriculum (e.g., Posner, 1992; Palomba and Banta, 1999). The *explicit curriculum* (EC) consists of the information in courses, textbooks, and workshops that are formally provided to learners. The EC contains the core concepts, norms, and values of an academic discipline. In that the content of the curriculum is clearly specified, the EC requires that learners achieve mastery of these theories, methods, and analytic skills. The *implicit curriculum* (IC) consists of the information that learners acquire throughout their studies that is not included in the explicit curriculum. The IC contains information that qualifies the formal curriculum such as exceptions to rules, as well as tacit knowledge or craft skills (e.g., Polanyi, 1958; Latour and Woolgar, 1979; Charlesworth et al., 1989). Craft skills can include how to select the “right” research question, how to effectively use experimental, analytic, and graphical software, and how to frame publications for acceptance. In that the IC is *ad hoc*, it can be considered an apprenticeship that relies on the specific knowledge and attention given to the learner by supervisors, mentors, and instructors.

## The standards of psychology

Key issues in scientific integrity have been outlined by governmental and non-governmental organizations in North America. In the United States, governmental standards have been provided by the Office of Research Integrity in terms of the responsible conduct of research (Steneck, 2006). In Canada, major granting organizations have provided general standards that are conditions of receiving research funds (Tri-council, 2006). University policies often extend these general standards but are highly variable in terms of their content (e.g., Greene et al., 1985; Lind, 2005; Schoenherr and Williams-Jones, 2011). For instance, while data falsification is universally agreed upon as deviant behavior, publication practices are not addressed to the same extent. Explicit and implicit curricula are left to address these concerns.

Professional organizations such as the American Psychological Association (APA) provide standards for responsible research practices to their members. In addition to the five general principles of conduct (beneficence and non-maleficence, fidelity and responsibility, integrity, justice, and respect for people's rights and dignity), the APA also identifies 10 guidelines related to scientific integrity (Section 8.10a to 8.15; American Psychological Association, 2002/2010). These APA guidelines address the reporting of research results (fabrication and error correction), plagiarism, publication credit (inclusion criteria, contributions, and student credit), duplicate publication, data sharing (post-publication and limitations), and roles and responsibilities of reviewers. Whether, and how, these norms are presented within the curriculum is an open question that I will briefly consider below.

## Undergraduate curriculum

The dearth of research on issues related to scientific integrity in psychological science can be contrasted with repeated reviews of the psychology curriculum more generally (Henry, 1938; Sanford and Fleishman, 1950; Daniel et al., 1965; Kulik, 1973; Lux and Daniel, 1978; Scheirer and Rogers, 1985; Cooney and Griffith, 1994; Perlman and McCann, 2005). For instance, Perlman and McCann (1999a) sampled the course catalogs of 400 institutions (evenly split among doctoral, comprehensive, baccalaureate, and 2-year colleges) from 1961 to 1997 to examine the courses offered by psychology departments. Courses that are provided across institutions provide insight into the family resemblance structure of the explicit scientific integrity curriculum within psychological science. If scientific integrity is viewed in terms of appropriate conduct through the planning, implementation, analysis, interpretation, and communication of research findings, identifying courses that likely contain this information provides a test for how scientific integrity is presented (Ellis, 1992). Thus, courses addressing experimental design, research methods, statistics, tests and measurement, as well as field experience represent an important starting point for queries into the scientific integrity curriculum (see Table 1).

**Table 1:** **Percentage of institutions sampled by Perlman and McCann (1999a) that offer undergraduate curriculum related to scientific integrity either for all institutions sampled (All) and the subsample of doctoral institutions (DU)**.

**Course related to scientific integrity**	**Year of university samples**								
	**1961**		**1969**		**1975**		**1997**		**DU Δ(97–75)**
	**All**	**DU**	**All**	**DU**	**All**	**DU**	**All**	**DU**	
Tests and measurement	48	84	48	81	51	**79**	51	**68**	**−11**
Statistics	36	74	43	75	46	**70**	48	**72**	**+2**
Experimental	45	84	45	73	39	**50**	44	**57**	**+7**
Field experience	–	–	–	–	30	**32**	30	**44**	**+12**
Research methods	–	–	–	–	42	**34**	42	**58**	**+24**

*Bold numbers reflect values used to obtain difference score. Difference score reflects change in course requirements from 1975 to 1997*.

Perlman and McCann's (1999a) study can be interpreted as evidence that scientific integrity-related courses were recurrent features of the psychology curriculum in most academic institutions. However, whether we consider all academic institutions that were sampled or solely doctoral institutions, it is clear that if these courses address scientific integrity issues then these issues might only be addressed in an inconsistent manner. In a follow-up study conducted by Perlman and McCann (2005), they also observed that courses that provide students with research experience were not obligatory and that there was considerable interdepartmental variability in terms of when these courses were offered. This underscores the importance of considering degree requirements.

A stronger test of the scientific integrity curriculum is to consider courses that are included in students' degree requirements. In a companion analysis of the structure of degrees in psychological science, Perlman and McCann (1999b) consider what courses were listed as degree requirements in 500 institutions. The majority of institutions listed capstone (i.e., courses that require integrating knowledge of theory and methods; 63%) and statistics (58%) as requirements while research methods courses (40%) and experimental psychology (38%) were required to a lesser extent. Other courses related to scientific integrity such as psychometrics (9%) and experimental design (7%) were degree requirements in the minority of institutions. In a comparable manner to their study of courses offered by institutions (Perlman and McCann, 1999a), Perlman and McCann (1999b) also note that degree requirements differed based on the type of institutions. For instance, whereas the majority of doctoral institutions required statistics courses (65%), comprehensive (59%) and baccalaureate institutions (49%) did so to a lesser extent. The variability in courses offered (Perlman and McCann, 1999a) and the extent to which they constitute degree requirements (Perlman and McCann, 1999b) suggest that the EC might only weakly addresses issues of scientific integrity.

Further support for curriculum variability is evidenced in the contents of research methods textbooks. Textbooks are a means to present ideal disciplinary standards in terms of core theories and evidence (e.g., Ash, 1983; Weiten and Wight, 1992; Zechmeister and Zechmeister, 2000). While research methods textbooks typically discuss issues of design (e.g., distinguishing between dependent and independent variables, participant selection, within-, or between-subjects design), scientific integrity issues (e.g., conflict of interests, data fabrication, publication practices) might not be included. Importantly, while research *ethics* is a near ubiquitous feature in research methods textbooks, these issues are restricted to the treatment of human and non-human participants. Although, research methods textbooks have begun to discuss misconduct, the minority mention the APA guidelines that address scientific integrity. For instance, an examination of a sample of research methods textbooks used at the author's institution (e.g., Smith and Davis, 2003; Shaughnessy et al., 2006; McBurney and White, 2010; Cozby and Rawn, 2012; Gravetter and Forzano, 2012; Leary, 2012) revealed that no textbook included all of these guidelines and that there is considerable variability in how many are discussed. While fabrication, error correction, and plagiarism were the most common forms of misconduct discussed, various aspects of publication credit and data sharing were addressed to a lesser extent. As with the EC, research methods textbooks used to support these courses do not appear to address the issues of scientific integrity in a comprehensive or consistent manner. This leaves the responsibility for scientific integrity education in the hands of individual instructors, supervisors, and mentors.

## Early experiences and graduate mentorship

It might be argued that many undergraduates neither necessarily seek, nor are considered for, graduate studies. Consequently, evaluation of the undergraduate psychology curriculum might not be the best approach to examining scientific integrity issues. For instance, while graduate and post-graduate researchers are concerned with research and publication, undergraduates need not be instructed in the specific practices required to conduct research. This argument reflects specious reasoning. First, higher education is directed toward understanding a research area. Researchers must understand the basic theories, experimental methods, and analytic procedures they use directly or indirectly (e.g., Schoenherr and Hamstra, 2015). This will minimally make students better consumers of scientific knowledge. Second, both socialization and expertise development require repeated use of social conventions, declarative knowledge, and technical skill. Given graduate students' first experience with responsible research practices occurs within the undergraduate curriculum, setting an early precedent is necessary. Moreover, as Lovitts (2007) has noted in the context of the doctoral dissertation, explicit conventions are often absent or not communicated to students. Michell (1997) goes further to claim that “many psychological researchers are ignorant with respect to the methods they use…the ignorance I refer to is about the *logic* of methodological practices,” (p. 356). If true, this suggests graduate school is not providing adequate instruction to develop these competencies.

A likely cause is revealed when we reflect on the experiences of graduate students. Much graduate work is based on self-directed learning. While courses are offered in advanced statistical techniques (e.g., multidimensional scaling, hierarchical linear modeling, factor analysis), other aspects of research methods are alluded to in research articles or left to supervisors and mentors to explicate. As research articles are a genre and limited in the extent to which they can discuss the research process, much of the scientific integrity curriculum is necessarily implicit. It is therefore likely to vary depending on the competency and experience of faculty members, reinforcing the importance of mentorship in education in general (e.g., Bird, 2001; Paglis et al., 2006; Anderson et al., 2007) and psychology in particular (e.g., Cronan-Hillix et al., 1986; Clark et al., 2000; Forehand, 2008). Concerns over the sufficiency of this form of apprenticeship must be addressed.

Once apprenticeship is recognized as a central feature of graduate studies, the extent to which psychologists share beliefs about scientific integrity becomes a central concern. However, psychologists have been found to disagree over the priority of APA standards (Seitz and O'Neill, 1996; Hadjistavropoulos et al., 2002) and are inconsistent in their application (Williams et al., 2012). Similar results have been observed for issues of scientific integrity. Riordan et al. (1988) examined psychologists' perceptions of plagiarism and fabrication. They note that while fabrication was viewed as more detrimental to a researcher's career, psychologists believed that university action was more justified in cases of plagiarism. More recently, John et al. (2012) assessed the prevalence and perceptions of questionable research practices by psychologists. They found that the manipulation of results in an unplanned and unreported manner was a reasonably common practice while also being judged to be dishonest by psychologists.

## Conclusions

Psychology is no more susceptible to disagreement over its norms than any other science (Ioannidis, 2005; De Vries et al., 2006). Consequently, variability of the undergraduate and graduate curricula suggests that a more *explicit* treatment of scientific integrity issues should be pursued. Despite the possibility that undergraduate statistics and research methods courses might address some of these issues in a general manner, other topics are not likely to be addressed. This appears to be reflected in the variable content of research methods textbooks. If departments are unclear as to whether this is the case, tools such as curriculum matrices (e.g., Levy et al., 1999) can be used to formally evaluate the features of their curriculum. Curriculum matrices require that faculty members identify core topics that should be covered within a curriculum and assess which courses address this information. When course information is plotted on such a grid, gaps are revealed and can then be addressed. In conjunction with the standards of professional organizations, formal policies, and guidelines can also be developed to ensure greater consistency.

## Funding

This research was supported by funding from the Ottawa Hospital Research Institute.

### Conflict of interest statement

The author declares that the research was conducted in the absence of any commercial or financial relationships that could be construed as a potential conflict of interest.

## References

[B1] American Psychological Association. (2002/2010). Ethical principles of psychologists and code of conduct. Am. Psychol. 57, 1060–1073. 12613157

[B2] Anderson M. S. Horn A. S. Risbey K. R. Ronning E. A. De Vries R. Martinson B. C. (2007). What do mentoring and training in the responsible conduct of research have to do with scientists' misbehavior?: findings from a national survey of NIH-funded scientists. Acad. Med. 82, 853–860. 10.1097/ACM.0b013e31812f764c 17726390

[B3] Ash M. G. (1983). The self-presentation of a discipline: history of psychology in the United States between pedagogy and scholarship, in Functions and Uses of Disciplinary Histories, eds Graham L. Lepenies W. Weingart P. (Dordrecht: Reidel), 143–189.

[B4] Bird S. J. (2001). Mentors, advisors and supervisors: their role in teaching responsible research conduct. Sci. Eng. Ethics 7, 455–468. 10.1007/s11948-001-0002-1 11697001

[B5] Charlesworth M. Farrall L. Stokes T. Turnbull D. (1989). Life Among The Scientists. Melbourne: Oxford University Press.

[B6] Clark R. A. Harden S. L. Johnson W. B. (2000). Mentor relationships in clinical psychology doctoral training: results of a national survey. Teach. Psychol. 27, 262–268. 10.1207/S15328023TOP2704_04

[B7] Cooney B. R. Griffith D. M. (1994). The 1992.1993 Undergraduate Department Survey. Washington, DC: American Psychological Association.

[B8] Cozby P. C. Rawn C. D. (2012). Methods in Behavioural Research, Canadian Edition. McGraw-Hill Ryerson.

[B9] Cronan-Hillix T. Gensheimer L. K. Cronan-Hillix W. A. Davidson W. S. (1986). Students' views of mentors in psychology graduate training. Teach. Psychol. 13, 123–127. 10.1207/s15328023top1303_5

[B10] Daniel R. S. Dunham P. J. Morris C. J. Jr. (1965). Undergraduate courses in psychology; 14 years later. Psychol. Rec. 15, 25–31.

[B11] De Vries R. Andersen M. S. Martinson B. C. (2006). Normal misbehaviour: scientists talk about the ethics of research. J. Empir. Res. Hum. Res. Ethics 1, 43–50. 10.1525/jer.2006.1.1.43 16810336 PMC1483899

[B12] Ellis H. C. (1992). Graduate education in psychology: past, present, and future. Am. Psychol. 47, 570–576. 10.1037/0003-066X.47.4.570

[B13] Forehand R. L. (2008). The art and science of mentoring in psychology: a necessary practice to ensure our future. Am. Psychol. 63, 744–755. 10.1037/0003-066X.63.8.744 19014235

[B14] Gravetter F. J. Forzano L. B. (2012). Research Methods for the Behavioral Sciences, 4th Edn. Belmont, CA: Wadsworth Cengage Learning.

[B15] Greene P. J. Durch J. S. Horwitz W. Hooper V. (1985). Policies for responding to allegations of fraud in research. Minerva 23, 203–215. 10.1007/BF01099942 11620773

[B16] Hadjistavropoulos T. Malloy D. C. Sharpe D. Green S. M. Fuchs-Lacelle S. (2002). The relative importance of the ethical principles adopted by the American Psychological Association. Can. Psychol. 43, 254–259. 10.1037/h0086921

[B17] Henry E. R. (1938). A survey of courses in psychology offered by undergraduate colleges of liberal arts. Psychol. Bullet. 35, 430–435. 10.1037/h0059009

[B18] Ioannidis J. P. A. (2005). Why most published research findings are false. PLoS Med. 2:e124. 10.1371/journal.pmed.0020124 16060722 PMC1182327

[B19] John L. K. Loewenstein G. Prelec D. (2012). Measuring the prevalence of questionable research practices with incentives for truth telling. Psychol. Sci. 23, 524–532. 10.1177/0956797611430953 22508865

[B20] Kulik J. A. (1973). Undergraduate Education in Psychology. Washington, DC: American Psychological Association.

[B21] Latour B. Woolgar S. (1979). Laboratory Life: The Construction of Scientific Facts. Princeton: Princeton University Press.

[B22] Leary M. R. (2012). Introduction to Behavioral Research Methods, 6th Edn. New York, NY: Pearson.

[B23] Levy J. Burton G. Mickler S. Vigorito M. (1999). A curriculum matrix for psychology program review. Teach. Psychol. 26, 291–294.

[B24] Lind R. A. (2005). Evaluating research misconduct policies at major research universities: a pilot study. Account. Res. 12, 241–262. 10.1080/08989620500217560 16634174

[B25] Lovitts B. E. (ed.). (2007). The psychology dissertation in Making the Implicit Explicit: Faculty's Performance Expectations for the Dissertation (Sterling, TX: Stlyus), 247–270.

[B26] Lux D. F. Daniel R. S. (1978). Which courses are most frequently listed by psychology departments? Teach. Psychol. 5, 13–16.

[B27] McBurney D. H. White T. L. (2010). Research Methods, 8th Edn. Belmont, CA: Wadsworth Cengage Learning.

[B28] Michell J. (1997). Quantitative science and the definition of *measurement* in psychology. Br. J. Psychol. 88, 355–383. 10.1111/j.2044-8295.1997.tb02641.x

[B29] Paglis L. L. Green S. G. Bauer T. N. (2006). Does adviser mentoring add value? A longitudinal study of mentoring in doctoral student outcomes. Res. High. Educ. 47, 451–476. 10.1007/s11162-005-9003-2

[B30] Palomba C. Banta T. (1999). Curriculum Assessment Essentials: Planning, Implementing & Improving Assessment in Higher Education. San Francisco, CA: Jossey-Bass.

[B31] Pashler H. Harris C. R. (2012). Is the replicability crisis overblown? Three arguments examined. Perspect. Psychol. Sci. 7, 531–536. 10.1177/1745691612463401 26168109

[B32] Perlman B. McCann L. I. (1999a). The most frequently listed courses in the undergraduate psychology curriculum. Teach. Psychol. 26, 177–182.

[B33] Perlman B. McCann L. I. (1999b). The structure of the psychology undergraduate curriculum. Teach. Psychol. 26, 171–176.

[B34] Perlman B. McCann L. I. (2005). Undergraduate research experiences in Psychology: a national study of courses and curricula. Teach. Psychol. 32, 5–14. 10.1207/s15328023top3201_2

[B35] Polanyi M. (1958). Personal Knowledge: Toward a Post-Critical Philosophy. Chicago, IL: University of Chicago Press.

[B36] Posner G. J. (1992). Analyzing the Curriculum. New York, NY: McGraw-Hill, Inc.

[B37] Riordan C. A. Marlin N. A. Gidwani C. (1988). Accounts offered for unethical Research practices: effects on the evaluations of acts and actors. J. Soc. Psychol. 128, 495–505. 10.1080/00224545.1988.9713769

[B38] Sanford F. H. Fleishman E. A. (1950). A survey of undergraduate psychology courses in American colleges and universities. Am. Psychol. 5, 33–37.

[B39] Scheirer C. J. Rogers A. M. (1985). The Undergraduate Psychology Curriculum: 1984. Washington, DC: American Psychological Association.

[B40] Schoenherr J. R. Hamstra S. J. (2015). Psychometrics and its discontents: a historical perspective on the discourse of the measurement tradition. Advan. Health Sci. Educ. [Epub ahead of print]. 10.1007/s10459-015-9623-z 26303112

[B41] Schoenherr J. Williams-Jones B. (2011). Research integrity/misconduct policies of Canadian universities. Can. J. High. Educ. 41, 1–17.

[B42] Seitz J. O'Neill P. (1996). Ethical decision-making and the code of ethics of the Canadian Psychological Association. Can. Psychol. 37, 21–30. 10.1037/0708-5591.37.1.23 11654993

[B43] Shaughnessy J. J. Zechmeister E. B. Zechmeister J. S. (2006). Research Methods in Psychology, 7th Edn. New York, NY: McGrawHill.

[B44] Simmons J. P. Nelson L. D. Simonsohn U. (2011). False-positive psychology: undisclosed flexibility in data collection and analysis allows presenting anything as significant. Psychol. Sci. 22, 1359–1366. 10.1177/0956797611417632 22006061

[B45] Smith R. A. Davis S. F. (2003). The Psychologist as Detective, 3rd Edn. Upper Saddle River, NJ: Pearson Prentice Hall.

[B46] Steneck N. (2006). Fostering integrity in research: definitions, current knowledge, and future directions. Sci. Eng. Ethics 12, 53–74. 10.1007/s11948-006-0006-y 16501647

[B47] Tri-council (2006). Tri-Council Policy Statement: Integrity in Research and Scholarship. Available online at: http://www.nserc.gc.ca/professors_e.asp?nav=profnav&lbi=p9

[B48] Weiten W. Wight R. D. (1992). Portraits of a discipline: an examination of introductory psychology textbooks in America, in Teaching Psychology in America: A History, eds Puente A. E. Matthews J. R. Brewer C. L. (Washington, DC: American Psychological Association), 453–504.

[B49] Williams J. Hadjistavropoulos T. Malloy D. C. Gagnon M. Sharpe D. Fuchs-Lacelle S. (2012). A mixed methods investigation of the effects of ranking ethical principles on decision making: implications for the canadian code of ethics for psychologists. Can. Psychol. 53, 204–216. 10.1037/a0027624

[B50] Zechmeister J. S. Zechmeister E. B. (2000). Introductory textbooks and psychology's core concepts. Teach. Psychol. 27, 6–11. 10.1207/S15328023TOP2701_1

